# Evidence of a high incidence of subclinically affected calves in a herd of cattle with fatal cases of Bovine Neonatal Pancytopenia (BNP)

**DOI:** 10.1186/s12917-014-0245-0

**Published:** 2014-10-08

**Authors:** Charlotte R Bell, Morag G Kerr, Philip R Scott, W Ivan Morrison, Helen Brown

**Affiliations:** The Roslin Institute and Royal (Dick) School of Veterinary Studies, University of Edinburgh, Easter Bush, Midlothian, Edinburgh, EH25 9RG Scotland UK; SAC Consulting: Veterinary Services, Edinburgh Disease Surveillance Centre, Allan Watt Building, Bush Estate, Penicuik, EH26 0QE Scotland UK; Division of Veterinary Clinical Studies, Royal (Dick) School of Veterinary Studies, University of Edinburgh, Easter Bush, Midlothian, Edinburgh, EH25 9RG UK

**Keywords:** Bovine Neonatal Pancytopenia (BNP), Haematology, Subclinical, Alloantibody

## Abstract

**Background:**

Bovine Neonatal Pancytopenia (BNP) is a disease of calves characterised by bone marrow trilineage hypoplasia, mediated by ingestion of alloantibodies in colostrum. Suspected subclinical forms of BNP have been reported, suggesting that observed clinical cases may not represent the full extent of the disease. However to date there are no objective data available on the incidence of subclinical disease or its temporal distribution. This study aimed to 1) ascertain whether subclinical BNP occurs and, if so, to determine the incidence on an affected farm and 2) determine whether there is evidence of temporal clustering of BNP cases on this farm. To achieve these aims, haematological screening of calves born on the farm during one calving season was carried out, utilising blood samples collected at defined ages. These data were then analysed in comparison to data from both known BNP-free control animals and histopathologically confirmed BNP cases. An ordinal logistic regression model was used to create a composite haematology score to predict the probabilities of calves being normal, based on their haematology measurements at 10–14 days old.

**Results:**

This study revealed that 15% (21 of 139) of the clinically normal calves on this farm had profoundly abnormal haematology (<5% chance of being normal) and could be defined as affected by subclinical BNP. Together with clinical BNP cases, this gave the study farm a BNP incidence of 18%. Calves with BNP were found to be distributed throughout the calving period, with no clustering, and no significant differences in the date of birth of cases or subclinical cases were found compared to the rest of the calves. This study did not find any evidence of increased mortality or increased time from birth to sale in subclinical BNP calves but, as the study only involved a single farm and adverse effects may be determined by other inter-current diseases it remains possible that subclinical BNP has a detrimental impact on the health and productivity of calves under certain circumstances.

**Conclusions:**

Subclinical BNP was found to occur at a high incidence in a herd of cattle with fatal cases of BNP.

## Background

Bovine Neonatal Pancytopenia (BNP) is a recently emerged disease of calves that has been described across Europe since 2007 [1] and in New Zealand since 2011 [2]. It presents as a bleeding disorder of calves less than one month old resulting in a high level of mortality in clinically affected calves [1,3] due to bone marrow trilineage hypoplasia [4] accompanied by depletion of peripheral thrombocytes and leukocytes. The disease is mediated by ingestion of alloantibodies in colostrum from particular cows [5,6] and the subsequent binding of these alloantibodies to calf hematopoietic cells [7,8], leading to functional compromise of bone marrow hematopoietic progenitors [9]. BNP is strongly associated with the use of a particular Bovine Viral Diarrhoea Virus (BVDV) vaccine (Pregsure BVD; Pfizer Animal Health) in the dams of affected calves [10,11] suggesting this vaccine induces alloantibody production in BNP dams. Furthermore, antibodies in serum from BNP-dams have been shown to react with the bovine kidney cell line used to generate the viral antigen incorporated in Pregsure [9] and also to recognise bovine MHC class I (MHC I) proteins [12,13] indicating that the stimulus for alloantibody production may be cell culture components within the vaccine.

Between March 2009 and February 2011 more than 4,500 confirmed cases of BNP were reported across Europe [14]. It is likely that this figure is an underestimate as considerable under-reporting of cases is known to occur [15] but nevertheless the incidence of clinical BNP on most affected farms is low, often with only one case per farm, rarely up to 10% [3,11]. These clinical cases may not represent the full extent of the disease, as suspected subclinical forms of BNP have been observed [3,16,17], where apparently healthy calves on BNP affected farms have been shown to have hematological profiles that appear abnormal. However, these findings were based on small numbers of animals of various/unspecified ages, in some cases with incomplete hematological data, and there has been no attempt to assess the extent of subclinical abnormalities in BNP-affected herds. Moreover, the precise definition of what constitutes an abnormal hematological profile in the affected age group of calves is currently problematic because of limited available information on the normal haemogram of calves in the postnatal period, which is known to differ significantly from adult cattle [18] and to change significantly with the age of the calf [19,20].

Temporal clustering of clinical BNP cases has been noticed on affected farms both within a single calving period [16,17] and seasonally [1,3,21]. Although this might be expected on dairy farms where colostrum is frequently pooled, such observations also come from beef herds where cross-suckling is unlikely. However thorough assessment of the temporal pattern of BNP requires inclusion of all calves affected by BNP, both clinical and subclinical, and therefore a clearer definition of all forms of the disease is necessary for appropriate analysis.

The aims of this study were 1) to ascertain whether subclinical BNP occurs and, if so, to determine the incidence on one affected farm and 2) to determine whether temporal clustering of BNP occurs on this farm. To achieve this, widespread haematological screening of calves was carried out on one BNP affected farm, with collection of samples at defined ages. These data were then analysed in comparison to both known BNP-free control animals and histopathologicically confirmed BNP cases. Furthermore, production data from the farm were analysed in order to assess the long-term impact of subclinical BNP on calves.

## Materials and methods

### Farms

The farm used for the investigation of subclinical BNP incidence (farm A) was a 200-cow, crossbred beef suckler herd, which had a 5% incidence of BNP in 2009 [16]. Sample collection on this farm took place during calving in spring 2010. Cows had been vaccinated with Pregsure BVD since 2007 and a booster vaccination was given in 2009, during the pregnancy from which calves were subsequently sampled. Throughout the study cows and calves were managed according to normal practice on that farm, with farm staff diligently ensuring prompt colostrum ingestion by calves. All calves suckled their own dams and no cross-suckling was observed. Calves were sired by natural service with ten bulls of three different breeds, providing a broad genetic background. Overall, during the 9 week peak period of spring calving samples were obtained from 143 calves from a total of 172 born (83%), and of these 4 showed clinical signs of BNP (see below).

Two BVDV-free and BVDV-unvaccinated dairy farms (farms B and C), with no known cases of BNP, supplied both calves and colostrum that served as controls.

### Calves

A summary of the groups of calves is provided in Table 1.

**Table 1:** **Summary of the groups of calves used in the study**

**Group**	**Number of Calves**	**BNP status**	**Farm of origin**	**BVDV vaccination status of dam**	**BVDV vaccination status of dam from which colostrum was ingested**	**Bone marrow histopathology**
**1**	139	Unknown – no clinical signs of BNP	A	Vaccinated	Vaccinated (own dam)	No
**2a**	14	BNP (Field cases)	A and similar local farms	Vaccinated	Vaccinated (own dam)	Yes
**2b**	5	BNP (Experimentally induced disease [22])	B and C	Unvaccinated	Vaccinated (not own dam)	Yes
**3a**	8	Unaffected normal	A	Vaccinated	Unvaccinated (not own dam)	No
**3b**	7	Unaffected normal	A	Unvaccinated	Unvaccinated (own dam)	No
**3c**	5	Unaffected normal (Experimental controls [22])	B and C	Unvaccinated	Unvaccinated (not own dam)	Yes

#### Calves with no clinical signs of BNP (Group 1, n = 139)

These comprised calves that showed no clinical signs of BNP, born during 2010 to dams on farm A with no history of producing a BNP affected calf. All calves were observed daily by a veterinary surgeon for the first 10–14 days of life, and daily thereafter by the farmer, and any calves that appeared abnormal were given a full clinical examination for signs indicative of BNP.

#### BNP cases (Group 2, total n = 19)

These comprised calves with clinical signs of BNP, born to dams on farm A in 2010 (n = 4) and 2009 (n = 4), and born to dams on five similar local farms in 2009 (n = 6). Of these 14 field cases (group 2a), 3 survived, while the remaining 11 fatal cases were confirmed by bone marrow histopathology. All these field cases showed typical clinical signs of BNP on examination by a veterinary surgeon. In addition, data was included from cases of BNP induced experimentally by feeding colostrum from BNP-dams on farm A to calves from farms B and C (group 2b, n = 5) [22]. These cases were euthanased at 10 days post-colostrum ingestion and in all cases bone marrow histopathology showed lesions consistent with BNP. Together these cases represent a broad range of severity of BNP that is typical of what is seen in the field.

#### Normal calves (Group 3, n = 20)

Calves that had been fed only colostrum from Pregsure unvaccinated cows were considered normal, as they were not exposed to any known potential source of BNP. These comprised two groups of calves from farm A; those that were born to BNP-dams in 2010 that were fed substitute colostrum from BVDV-unvaccinated cows (group 3a, n = 8) [23] and calves born in 2011 to dams that had not been vaccinated with Pregsure (group 3b, n = 7). In addition, data were included from experimental control calves from farms B and C (group 3c, n = 5) fed colostrum from cows on farms B and C [22]. None of these calves showed any clinical signs of BNP, and in the experimental calves bone marrow histopathology at necropsy at 10 days old was within normal limits.

### Blood sampling and haematology

For all calves a single blood sample was collected by jugular venepuncture at 10–14 days old and transferred immediately into a K-EDTA collection tube. Haematological parameters (erythrocyte, thrombocyte and total leukocyte counts) were measured with an automated haematology analyser (Beckman Coulter Ac. T5 impedance counter, Beckman Coulter, UK) and differential leukocyte counts were performed manually on a freshly prepared blood smear stained with a Romanowsky stain (Reastain Quick Diff, Reagena, Finland). Blood was processed within 24 hours of collection, a timeframe which, in contrast to adult cattle, has been demonstrated to provide a reproducible thrombocyte count in young calves [20], and no signs of thrombocyte clumping were observed on manual verification of a blood smear.

The 10–14 day time point was selected for investigation of subclinical BNP because, in BNP cases, it represents a nadir in haematological values and thus the period during which haematological abnormalities related to BNP might most easily be detected [5]. Moreover, for both normal calves and BNP, large changes in haematological parameters occur in the first week of life [18,22] and thus later sampling is less subject to such variation and allows a larger window for sample collection.

Samples were also collected at < 24 hours old from 136 calves in group 1, four calves in group 2a and all calves in groups 2b, 3a and 3c, and analysed using the same methods. Calves in group 2b and 3c were sampled on seven set occasions during the first 24 hours, all post-colostrum ingestion, and therefore a mean value was calculated for each parameter and used for further analysis. For all other groups only a single sample was collected during the first 24 hours irrespective of the time of colostrum ingestion.

### Statistical analysis of haematology results

Samples collected at 10–14 days were analysed first, and measurements from calves in group 2 (n = 19) were used to define the haematological profile of a BNP case and group 3 (n = 20) were used to define the haematological profile of a normal calf. Haematological parameters for these groups were compared using t-tests, when the logs of measurements were normally distributed, and Wilcoxon rank sum tests for other measurements (basophils and eosinophils), in order to determine which parameters differed significantly between the two groups. In addition, these tests were carried out separately for samples collected at < 24 hours after birth.

An ordinal logistic regression model was used to create a composite hematology score to predict probabilities of calves being normal, based on their haematology measurements at 10–14 days old. The disease status of calves was ordered as; BNP cases (group 2), calves with no clinical signs of BNP (group 1) and normal calves (group 3). Haematology measurements were entered into the model one-by-one (ie using forward selection) until no further measurements had a p-value of less than 0.20. Natural logarithms of measurements were used when these provided a more symmetrical distribution (all measurements except basophils and eosinophils); otherwise untransformed values (basophils and eosinophils) were used. Inclusion of the group 1 calves in this analysis allowed the model to utilise the partial information on disease status (ie. not clinical BNP but not known whether normal) available from this large group of calves. The coefficients from the logistic equation were used to form an additive score from the combined haematology measurements, and the distribution of scores obtained for the normal calves (group 3) was used to identify calves with low probability of being normal based on hematology measurements at 10–14 days. This was achieved by assuming a normal distribution for the scores of normal calves (group 3) and obtaining the 5% lower tail. Calves in group 1 with scores below this point were defined as affected by subclinical BNP.

Calves defined by the model as affected by subclinical BNP were then separated from the other calves in group 1. Individual haematology parameters at < 24 hours for the subclinical calves were compared to those in the remainder of group 1 using t-tests when the logs of measurements were normally distributed, and Wilcoxon rank sum tests for other measurements (basophils and eosinophils), in order to investigate whether subclinical calves showed significant alterations in haematology at < 24 hour old. Additionally mortality was compared between the subclinical calves and the remainder of group 1 using a Fisher’s exact test. Statistical analyses were performed using SAS/STAT® software and GenStat, VSN International, (version 13).

### Determining the temporal pattern of BNP

A Monte Carlo simulation approach was used to determine whether the birth dates of the four BNP cases observed on farm A in 2010, or the four BNP cases plus the subclinical BNP calves, were significantly clustered in time. First all possible clusters across time and with varying lengths of cluster period (4–14 days) were considered and the ‘best’ cluster with the highest likelihood was determined. To determine if the likelihood of this ‘best’ cluster was higher than expected by chance, the distribution of maximum likelihood values expected under no clustering was obtained using Monte Carlo simulation. First 1000 examples of daily case and control numbers occurring were simulated under the expectation of an even ratio cases/controls across time; the maximum likelihood for the ‘best’ clusters was then obtained for each simulated example (just as for the observed data); then these 1000 maximum likelihood values were used to provide the required ‘null’ distribution. The significance of the maximum likelihood value for the observed data was obtained by comparing it to this distribution.

Additionally, t-tests were used to compare the distribution of birth dates for BNP calves with both subclinical calves and the remainder of group 1, and to compare the distribution of birth dates of subclinical calves with the remainder of group 1.

### Calf production data

All calves from farm A were sold at final slaughter weight. Records of days from birth to sale were available for 127 of the 139 calves of unknown BNP status (group 1), and these were used as an indicator of production performance from birth to slaughter. To assess the impact of abnormal haematology at 10–14 days on subsequent production performance, days from birth to sale were analysed using a Cox regression model which fitted composite haematology score and date of birth (hence adjusting for any influence of time of birth on age at sale). Calves that died from non-BNP causes prior to sale were included as censored observations, and calves retained for breeding and not sold were excluded from the analysis.

### Ethical considerations

Blood sampling of calves from the BNP affected farm (farm A) was undertaken for veterinary disease investigation purposes after consultation with the UK Home Office and review by the Royal (Dick) School of Veterinary Studies Ethical Review Committee. Calves in groups 2b and 3c (experimentally induced BNP cases and experimental controls) were sampled as part of a study conducted at the Moredun Research Institute, UK. These calves were managed in accordance with the UK Animals (Scientific Procedures) Act 1986 and the experiment was approved by the Moredun Ethical Review Committee (experiment number 51/10).

## Results

### Analysis of haematology parameters at 10–14 days old

A summary of the haematology results obtained at 10–14 days old for the three groups of calves is provided in Table 2. In samples taken at 10–14 days old, haematology parameters in case calves (group 2) were significantly lower than those in normal calves (group 3) for thrombocytes (p < 0.001), monocytes (p < 0.002), erythrocytes (p < 0.001), lymphocytes (p < 0.001), neutrophils (p < 0.001), band neutrophils (p = 0.03), and basophils (p = 0.05), in accordance with other studies [22]. However, the significance of band neutrophils and basophils should be considered borderline given the multiple tests carried out. There was no significant difference between the groups for eosinophils (p = 0.61).

**Table 2:** **Summary haematology results and composite haematology scores obtained for each group of calves**

**Haematology results at 10–14 days**	**Calves with no clinical signs of BNP (Group 1)**	**Clinical BNP cases (Group 2)**	**Normal calves (Group 3)**	**Calves from group 1 identified by the models as affected by subclinical BNP**
Erythrocytes (×10^12^/L)	7.69 (6.35-9.03)	5.87 (3.87-7.87)	8.35 (6.96-9.74)	7.77 (6.31-9.23)
Thrombocytes (×10^9^/L)	812 (476–1148)	52 (0–140)	844 (624–1064)	401 (192–610)
Total leukocytes (×10^9^/L)	11.64 (7.86-15.42)	2.53 (0.83-4.23)	13.60 (8.35-18.85)	7.64 (4.50-10.78)
Segmented neutrophils (x10^9^/L)	6.96 (3.73-10.19)	1.28 (0–3.09)	7.07 (2.51-11.63)	5.19 (2–8.38)
Band neutrophils (×10^9^/L)	0.07 (0–0.28)	0.02 (0–0.07)	0.30 (0–0.83)	0.02 (0–0.07)
Lymphocytes (×10^9^/L)	4.19 (2.45-5.92)	1.17 (0–2.05)	4.84 (2.92-6.76)	2.25 (1.46-3.04)
Monocytes (×10^9^/L)	0.35 (0–0.76)	0.02 (0–0.06)	0.86 (0–1.89)	0.11 (0–0.27)
Eosinophils (×10^9^/L)	0.04 (0–0.15)	0.02 (0–0.09)	0.04 (0–0.12)	0.08 (0–0.23)
Basophils (×10^9^/L)	0.02 (0–0.07)	0.00 (0–0)	0.09 (0–0.33)	0.00 (0–0.03)
**Composite Haematology Score**	**13.17 (1.52)**	**4.55 (2.71)**	**14.87 (1.94)**	
**Haematology results at <24 hours**	**Calves with no clinical signs of BNP (Group 1)**	**Clinical BNP cases (Group 2)**	**Normal calves (Group 3)**	**Calves from group 1 identified by the models as affected by subclinical BNP**
Erythrocytes (×10^12^/L)	8.75 (7.41-10.10)	8.37 (6.99-9.76)	8.92 (7.06-10.78)	9.13 (7.86-10.41)
Thrombocytes (×10^9^/L)	375 (272–478)	335 (272–398)	504 (239–769)	292 (169–416)
Total leukocytes (×10^9^/L)	12.16 (6.32-17.99)	9.86 (5.46-14.27)	16.50 (8.34-24.66)	8.82 (4.36-13.28)
Segmented neutrophils (×10^9^/L)	9.12 (4.18-14.06)	7.27 (4.27-10.26)	12.58 (5.99-19.16)	6.83 (2.75-10.92)
Band neutrophils (×10^9^/L)	0.63 (0–1.87)	0.93 (0.26-1.60)	0.87 (0.13-1.62)	0.44 (0–0.91)
Lymphocytes (×10^9^/L)	2.12 (0.9-3.35)	1.54 (0–3.07)	2.72 (1.16-4.27)	1.39 (0–2.88)
Monocytes (×10^9^/L)	0.20 (0–0.43)	0.05 (0.01-0.10)	0.25 (0.04-0.45)	0.08 (0–0.21)
Eosinophils (×10^9^/L)	0.06 (0–0.21)	0.05 (0–0.12)	0.03 (0–0.11)	0.04 (0–0.14)
Basophils (×10^9^/L)	0.02 (0–0.09)	0.00 (0–0)	0.04 (0–0.14)	0.01 (0–0.05)

Mean values are shown, with one standard deviation above and below the mean in parentheses.

The results of the ordinal logistic regression analysis are shown in Table 3. The following composite haematology score was obtained using the coefficients of this model and used to predict the likelihood of individual calves being affected by BNP based on measurements taken at 10–14 days after birth:

**Table 3:** **Results of the ordinal logisitic regression analysis**

	**Estimate (SE) (on logit scale)**	**p-value**
Intercept 1	−16.03 (2.82)	<0.0001
Intercept 2	−8.03 (2.12)	0.0002
Log(Monocytes +1) (x10^9^/L)	1.46 (0.92)	0.11
Log(Lymphocytes +1) (x10^9^/L)	2.52 (0.75)	0.0008
Log(Band neutrophils + 1) (x10^9^/L)	3.59 (1.65)	0.03
Log(Thrombocytes + 1) (x10^9^/L)	1.29 (0.39)	0.001
Basophils (x10^9^/L)	4.64 (2.58)	0.07

$$ \begin{array}{l}\mathrm{Score} = 1.46\ \mathrm{x}\  \log \left(\mathrm{monocytes}+1\right) + 2.52\ \mathrm{x}\  \log \left(\mathrm{lymphocytes}+1\right) + 1.29\ \mathrm{x}\  \log \left(\mathrm{thrombocytes}+1\right)\ \\ {} + 3.59\ \mathrm{x}\  \log \left(\mathrm{band}\ \mathrm{neutrophils}+1\right) + 4.64\ \mathrm{x}\ \mathrm{basophils}\end{array} $$

Means and standard deviations for the composite haematology scores for each group of calves are shown in Table 2. Thrombocytes and lymphocytes made the greatest contributions to the score when judged by the coefficient sizes of a model equivalently defined in terms of standardised haematology values (ie divided by their standard deviations).

**Figure 1:**
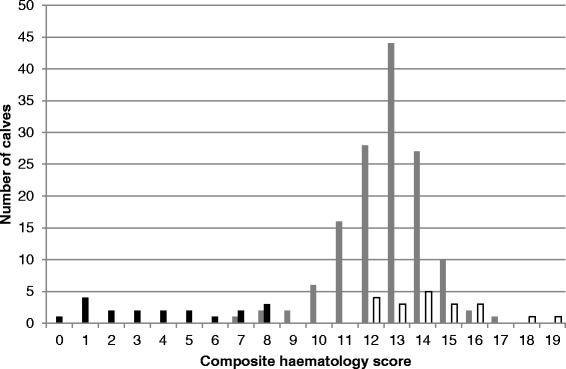
**Frequency of calves with different composite haematology scores (rounded to integer values).** Calves with no clinical signs of BNP (group 1) – grey columns, BNP cases (group 2) - black columns, normal calves (group 3) - white columns. Calves with a composite haematology score of <11.68 are predicted to have a less than 5% chance of being normal.

Overall the composite haematology scores for calves in the BNP-affected herd (group 1) were significantly lower (p < 0.001) than that of the normal calves (group 3) (t-test). The distributions of composite haematology scores for groups 1, 2 and 3 are shown in Figure 1.

### Determining the incidence of subclinical disease

Based on the distribution of scores of normal calves (group 3), calves with a composite haematology score of less than 11.68 were predicted to have a less than 5% chance of being normal. Fifteen percent (21 of 139) of the calves in group 1 that showed no overt signs of BNP had composite haematology scores below this value and therefore could be defined as being subclinically affected by BNP. Scores of the BNP cases (group 2) and normal calves (group 3) were completely separated and no normal calves had scores below the threshold used to define the subclinical calves, and no BNP cases had scores above it (Figure 1).

### Analysis of haematology parameters at <24 hours old

A summary of the haematology results obtained at <24 hours old for the three groups of calves, is provided in Table 2. In samples taken at < 24 hours old, haematology parameters in case calves (group 2) were significantly lower than those in normal calves (group 3) for monocytes (p = 0.005), thrombocytes (p = 0.05) and neutrophils (p = 0.02). There was no significant difference between the groups for lymphocytes (p = 0.1), erythrocytes (p = 0.23), band neutrophils (p = 0.57), eosinophils (p = 0.49) and basophils (p = 0.20), although statistical power of these tests was low with only 9 BNP cases and 13 normal calves available. Furthermore the significance of thrombocytes and neutrophils should be considered borderline given the multiple tests carried out.

At <24 hours old, calves subsequently identified by the haematology score at 10–14 days as affected by subclinical BNP had significantly lower haematological parameters than the remainder of calves in group 1 for monocytes (p < 0.001), thrombocytes (p < 0.001), lymphocytes (p = 0.002) and neutrophils (p = 0.02), but not for erythrocytes (p = 0.16), band neutrophils (p = 0.17), eosinophils (p = 0.55) and basophils (p = 0.75) (see Table 2). Again the significance of neutrophils should be considered borderline given the multiple tests carried out. These results suggest that the calves identified by the haematology score as affected by subclinical BNP have significant alterations in haematology in the first 24 hours of life and the observed pattern of alterations in individual haematology parameters is consistent with that observed in BNP cases used in this study (group 2). This finding is particularly notable given that samples were collected at a range of time-points following colostrum ingestion, which would be expected to result in variation on the severity of haematological alternations in individual calves [16].

### Assessing the long-term impact of subclinical BNP

Age at sale was associated with haematology score (p = 0.04) and date of birth (p = 0.006). Calves with lower scores and later dates of birth were significantly younger at sale. The mean age at slaughter in subclinical calves was 461 days, compared to the remainder of group 1 with a mean of 479 days. There was not a statistically significance difference in mortality between the subclinical calves and the remainder of group 1 (p = 0.6). While there was limited power to assess this difference, the lower mortality rate of 0% in the subclinical calves compared to 4.3% in the remainder of group 1 indicated that higher mortality in subclinical calves was unlikely.

### Determining the temporal pattern of BNP

**Figure 2:**
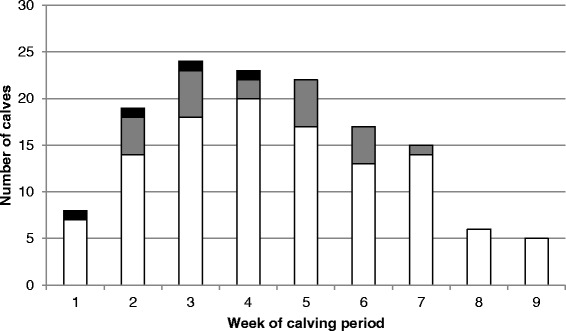
**Distribution of all calves sampled on farm A (n = 143) by week of calving.** Calves are divided into; BNP cases (n = 4, black columns), calves in group 1 identified as subclinical BNP cases by the model (n =21, grey columns) and the remaining calves from group 1 (n = 118, white columns).

The distribution of the normal calves, subclinical calves and BNP calves in this herd by week of birth is shown in Figure 2. While the mean days from the start of the calving period until birth was shorter in BNP cases (14 days) than subclinical cases (26 days) or calves in the remainder of group 1 (29 days), the differences were not significantly different (BNP cases compared to group 1, p = 0.07; BNP cases compared to subclinical cases, p = 0.06; subclinical cases compared to remainder of group 1, p = 0.40). There was also no significant clustering in birth dates of BNP cases, or BNP cases plus subclinical cases, in this herd during 2010.

## Discussion

This study demonstrates that approximately 15% of calves on a BNP affected farm had an abnormal haematology profile at 10–14 days of age, but without showing any overt clinical signs of BNP. The haematology parameters of these calves, although less severely affected than in BNP cases, displayed a profile that is similar to that of clinical cases. These findings, coupled with the absence of such animals in the control populations, indicate that these animals are likely to represent sub-clinical cases of BNP. Furthermore, in a separate study (manuscript in preparation) we have demonstrated that cows from this BNP-affected herd that produced calves without clinical signs of BNP have significantly higher titres of serum alloantibody than BVDV-unvaccinated cows, providing supporting immunological evidence for these epidemiological findings.

Together with the four clinical cases of BNP diagnosed on the study farm in 2010, this gives an overall incidence of BNP on this farm of 18%, with >5 subclinical cases of BNP for every case with overt clinical signs. As the study herd comprised cross-bred cows originating from across the UK, and did not use special colostrum management or colostrum pooling during the study period, these results suggest that the haematological manifestations of BNP are much more widespread than indicated by the incidence of overt clinical disease. However, as this study was conducted on a single BNP-affected herd, caution must be exercised in generalising these results to the wider cattle population with differing management systems and genetic backgrounds and therefore further studies on other herds using the model described would be informative.

These findings can also be used to make inferences about the proportion of cows producing BNP alloantibody. When calves are fed colostrum from individual unrelated BNP-dams a proportion (roughly 1 in 3) do not succumb to disease [6] presumably because they lack antigens that are sufficiently complementary to the specific alloantibodies generated by that cow. When calves suckle their own dams the proportion of calves unaffected by BNP is likely to be even higher, as half their genetic material will originate from the dam, reducing the likelihood of susceptible antigens being present. The same rationale is likely to apply to subclinical BNP, and thus the percentage of cows producing BNP alloantibodies in this affected herd is likely to be higher than 18%. This is supported by studies investigating the implicated anti-bovine MHC I antibodies, which have suggested that 50-100% of cows vaccinated with Pregsure following the recommended protocol may produce these antibodies [12,24].

Results from this study also demonstrate that BNP is not an all-or-nothing response but rather that a continuum of disease exists. Variation in BNP severity, from clinical to subclinical, could be influenced by differences in the quantity and alloantibody titre of ingested colostral antibody and the time from birth to suckling, all of which will influence the amount of alloantibody passively transferred to the calf. In addition, a range of disease severity is consistent with evidence that alloantibodies produced by BNP dams are specific for highly polymorphic antigens [8,12,13] which would be likely to display a complex pattern of antigenic specificities leading to variable affinity to calf cells of differing genotypes.

This study failed to find significant temporal clustering of clinical or subclinical BNP or significant differences in the date of birth of cases or subclinical cases compared to the rest of the calves. During this study colostrum pooling was not practised and calves were housed and managed in a similar manner for the duration of the sampling period. Previous reports of a temporal pattern in the occurrence of BNP cases [3,16,25] might therefore relate to pooling of colostrum or variation in management factors during the calving period which increase the likelihood of BNP manifesting clinically. For example, concurrent disease or mild trauma due to handling or crowding could increase the likelihood of subclinical cases manifesting clinically and might also explain the reduced risk of BNP in calves kept outdoors [10]. As all dams in this study were vaccinated with Pregsure at a single time point this result also supports previous findings of no association between the incidence of BNP and the time since last Pregsure vaccination [10].

In this study, subclinical BNP did not increase calf mortality or have a detrimental effect on long-term production in terms of days to slaughter. However other studies looking at larger numbers of farms with varied management conditions have shown an increase in calf morbidity attributed to causes other than BNP on affected farms, since the appearance of the disease [15]. A positive association has also been found between BNP affected farms and the incidence of calf respiratory disease and treatments for neonatal calf diseases [11,15]. Lack of clinical signs of bleeding in subclinical cases of BNP is consistent with redundancy of haemostatic mechanisms which mean that only profound thrombocytopenia (often <30×10^9^/L) will lead to bleeding diathesis [26]. However profoundly abnormal haemograms due to subclinical BNP, which include depressed lymphocyte and monocyte counts, may result in immunosuppression, providing a credible basis for the suggestion that subclinical BNP could have an impact on calf health and productivity in situations where animals are exposed to pathogen challenge.

## Conclusions

This study has demonstrated that on one BNP affected farm 15% of clinically normal calves had profoundly abnormal haematology and could be defined as affected by subclinical BNP. The haematology parameters of these calves, although less severely affected than in BNP cases, displayed a profile that is similar to that of clinical cases. Calves with BNP were found to be distributed throughout the calving period, with no clustering, and no significant differences in the date of birth of cases or subclinical cases were found compared the rest of the calves. This study did not find any evidence of increased mortality or increased time from birth to sale in subclinical BNP calves but, given that this study only involved a single farm, a detrimental impact of subclinical BNP on the health and productivity of calves cannot be ruled out.
